# Prognostic value of circulating endothelial cells in glioblastoma patients: a pilot study

**DOI:** 10.2144/fsoa-2022-0008

**Published:** 2022-05-24

**Authors:** María Angeles Vaz Salgado, Julie Earl, Victor Rodriguez Berrocal, Freddy Salge Arrieta, Ana Gomez, Juan Manuel Sepulveda-Sanchez, Ángel Perez-Nuñez, Elena Corral de la Fuente, Daniel Lourido, María Villamayor, Hector Pian, Alfonso Muriel, Elisabetta Rossi, Rita Zamarchi, Alfredo Carrato, Luis Ley

**Affiliations:** 1 Medical Oncology Department. Ramón y Cajal Hospital. IRYCIS, CIBERONC, Madrid, 28034, Spain; 2 Neurosurgery Department. Ramón y Cajal Hospital, Madrid, 28034, Spain; 3 Medical Oncology Department. 12 de Octubre Hospital. Instituto de Investigación I+12, Madrid, 28041, Spain; 4 Neurosurgery Department. 12 de Octubre Hospital, Madrid, 28034, Spain; 5 Radiology Department. Ramón y Cajal Hospital, Madrid, 28034, Spain; 6 Pathology Department, Ramón y Cajal Hospital, Madrid, 28034, Spain; 7 Clinical Biostatistic Unit, Hospital Universitario Ramón y Cajal, IRYCIS, CIBERESP, Nursing Department Universidad de Alcalá Madrid, 28034, Spain; 8 Department of Surgery, Oncology & Gastroenterology, University of Padova, Padua, 35124, Italy; 9 Veneto Institute of Oncology IOV – IRCCS, Padua, 35128, Italy

**Keywords:** biomarker, circulating endothelial cells (CECs), glioblastoma, perfusion MRI

## Abstract

**Aim:**

Glioblastoma (GB) is an aggressive tumor type and the detection of circulating endothelial cells (CECs) in peripheral blood has been related to angiogenesis.

**Materials & methods:**

A prospective single-center pilot study of CEC detection at diagnosis in 22 patients with GB was performed, using the US FDA-approved CellSearch system.

**Results:**

A CEC cutoff value was estimated using a receiver operating curve (ROC) and patients were classified into two groups: <40 CEC/4 ml and >40 CEC/4 ml blood. Median overall survival was 25.33 months for group 1 and 8.23 months for group 2 cases (p = 0.02). There was no correlation between CEC and PWI (perfusion-weighted imaging) RM.

**Conclusion:**

CEC detection has a prognostic value in GB cases at diagnosis.

## Plain language summary

Glioblastoma is an aggressive tumor type with a general poor prognosis. Endothelial cell detection in blood samples has been previously described as a marker of outcome in many tumor types. A US FDA-approved device, CellSearch, was used for CEC detection. The patients were split into two groups according to a cutoff value with the highest sensitivity and specificity, group 1 <40 and group 2 >40 CEC/4 ml blood. Patients with >40 CEC at diagnosis had a poorer overall survival; 25.33 months for group 1 and 8.23 months group 2 cases, which was statistically significant.

Glioblastoma (GB) is a highly aggressive tumor, with a median survival of approximately 15 months in spite of treatment with surgery, radiotherapy and temozolomide. The current standard of care is surgery, radiotherapy and concomitant temozolomide followed by adjuvant cycles of temozolomide for at least 6 months [1]. GB is a very vascularized tumor and prognosis is related with this high vascularity. In addition, there is a need for minimally invasive biomarkers to monitor disease evolution during the treatment of GB [2,3]. Angiogenesis in the primary tumor can lead to vascular damage and shedding of endothelial cells into the blood stream. Circulating endothelial cells (CEC) are increasingly considered with interest as predictive biomarkers, as diverse stimuli facilitate cell migration from the bone to complement local angiogenesis [4].

CEC are endothelial cells that are present in the peripheral circulation and include endothelial progenitor cells (CEPs) and mature endothelial cells shed from vessels walls and cells with an endothelial function from cancerous cells [5]. The mechanism of CEC shedding is not fully understood but is thought to be a consequence of physical damage to blood vessels, reduced adhesion and binding of cells with the basement membrane [6]. Therefore, CEC have been established as markers of endothelial damage or dysfunction and response to anti-angiogenic agents in various tumor types including breast, lung, colorectal and renal cancer [7,8,9,10]. CEC are rarely found in healthy individuals are usually found in individuals with disease such as COVID-19, blood vessel damage and cancer [6,8]. Furthermore, a recent study has shown that the ratio of viable: apoptotic CEC is an indicator of COVID-19 severity [9,10].

In cancer patients, the CEC number correlates with tumor progression and constitutes a promising tool to monitor disease activity, with the potential for the assessment of prognosis and response to treatment. In breast and lung cancer, an association has been observed between elevated CEC and longer progression free survival [8,11]. Previous reports established a correlation between CEP levels and tumor grade and prognosis in glioma patients [12,13].

Magnetic resonance imaging (MRI) is the gold standard imaging diagnosis for GB. Perfusion-weighted MRI (PWI) is a technique for the quantification of cerebral blood flow and volume (CBV). GB have been demonstrated to have regions of significantly elevated rCBV, consistent with their marked vascular proliferation [14].

This pilot study aimed to evaluate the potential prognostic value of CEC in patients with newly diagnosed GB in terms of survival. Levels of CEC, CEP, rCBV were determined in a cohort of patients with GB. These blood and RM-imaging biomarkers were hypothesized to be associated with a poorer prognosis.

## Materials & methods

### Patient selection

A prospective study was performed of patients with a newly diagnosed glioblastoma who received standard treatment at the Ramon y Cajal University Hospital in Madrid, Spain. The inclusion criteria were patients over 18 years of age, with a diagnosis of GB confirmed by histopathology. The patients were included between 2014 and 2016. Patients' characteristics are shown in Table 1. All patients with a suspected glioblastoma and those willing to participate in this study were considered, therefore the sample can be considered representative of a larger population. At the time of the study inclusion, the WHO classification of 2016 was not in clinical use.

**Table 1.** Patient characteristics.

Characteristics	Patients (n = 22)
Age, years (average)	63
Median age (range)	42–81
Sex	
Male	63.6%
Female	36.4%
ECOG (diagnosis)	
0	27.3%
1	68.2%
ECOG post-surgery	
0	22.7%
1	72.7%
3	4.5%
Location	
1 – frontal	9 (41%)
2 – temporal	9 (41%)
3 – parietal	2 (10%)
4 – occipital	1 (4%)
5 – others	1 (4%)
Extent of surgery	
Biopsy	18%
Partial and subtotal resection	32%
Complete resection	50%

The patients received standard treatment based on radiotherapy and temozolomide as defined by the Stupp regimen. Some patients did not receive telozolomide as they were fragile and aged patients. After surgery, they received radiotherapy plus concomitant temozolomide followed by adjuvant temozolomide. Patients were assessed by magnetic resonance imaging (MRI). At progression, some patients were amenable for second-line treatment. Progression-free survival (PFS) was defined as the time from the initial histological GB diagnosis to time of disease recurrence or progression, based on imaging. Overall survival (OS) was defined as the time from the initial histological diagnosis to death. The clinical data were obtained from the hospital medical records. As this study was as an exploratory analysis, the sample size was determined by the number of patients diagnosed during this period of time and those that chose to participate in the study.

### CEC detection

Peripheral blood samples were obtained at the time of diagnosis of glioblastoma. Endothelial cells were measured by the US FDA-approved CellSearch Assay^®^ in 4 ml of whole blood as CD146^+^, CD105^+^ CD45-DAPI+. A total of 22 patients with glioblastoma were analyzed.

### MRI of glioblastoma

The specific MRI protocols differed slightly between patients, however, they usually included T1-weighted pre- and post-contrast agent administration, fluid-attenuated inversion recovery, diffusion-weighted and T2-weighted scans. Dynamic susceptibility weighted was used for PWI, whereby dynamic MRIs were acquired after a bolus of contrast agent was administered intravenously. PWI was not performed in all patients as part of a standard brain tumor MRI protocol. For the quantification of the perfusion parameters, a ROI (region of interest) was drawn manually over the enhancing portions of the tumor, selecting visually those areas including the highest relative blood volume. Then the relative blood volume was calculated comparing the data obtained with the contralateral normal appearing white matter.

### Statistical analysis

Statistical analysis was performed using the SPSS and R software. ROC curves (receiver operating curve) were used to estimate the cutoff value for CEC detection, and the patients were classified into two groups depending on the average number of CEC for subsequent analysis: group 1 had an average CEC count below 40 and group 2 had an average CEC count of 40 or above. OS and PFS were analyzed by the Kaplan–Meier method and survival curves of the subgroups were compared using the log-rank test. A Spearman test was performed to determine the correlation between CEC and rCBV parameters in MRI and the Mann–Whitney test to compare CEC count before and after surgery/radiotherapy.

## Results

### Patient characteristics

Twenty-two patients were included between 2014 and 2016. All patients had histologically confirmed glioblastoma. There were 14 males and 8 females and the average age was 63 years (range: 42–81 years). ECOG was 0–1 in 95% of cases. A complete resection was achieved in 50% of cases, partial or subtotal resection in 31.8% and biopsy in 18%. The extension of the resection was defined according to the opinion of the surgeon. All patients received radiotherapy and 3/22 (13.6%) did not receive concurrent temozolomide. 9/22 (41%) patients were treated with adjuvant temozolomide for six or more cycles.

### Circulating endothelial cell detection

Determination of CEC was performed prior to the surgical procedure in 26 patients, 22 were high grade, recently diagnosed with glioblastoma. The average number of CEC was 59.3 cells/ml (range: 0–954) and 32% (7/22) patients were negative for CEC. The median number of CEC was 5 cell/4 ml. The sample cohort was divided into two groups according to the CEC count at diagnosis. The cutoff was calculated using the receiver operating curve (ROC) and the value with the highest sensitivity and specificity was selected. In this cohort, the cutoff value was 40 CEC/4 ml blood, HR 6.69 (IC: 1.72–25.92). Patients were classified into two groups: group 1 had a CEC count of ≤40 cells/ml and group 2 had a CEC count >40. A total of 17 patients (77.2%) were in group 1 and 5 patients (22.7%) were in group 2.

### CEC count & prognosis

**Figure 1.**
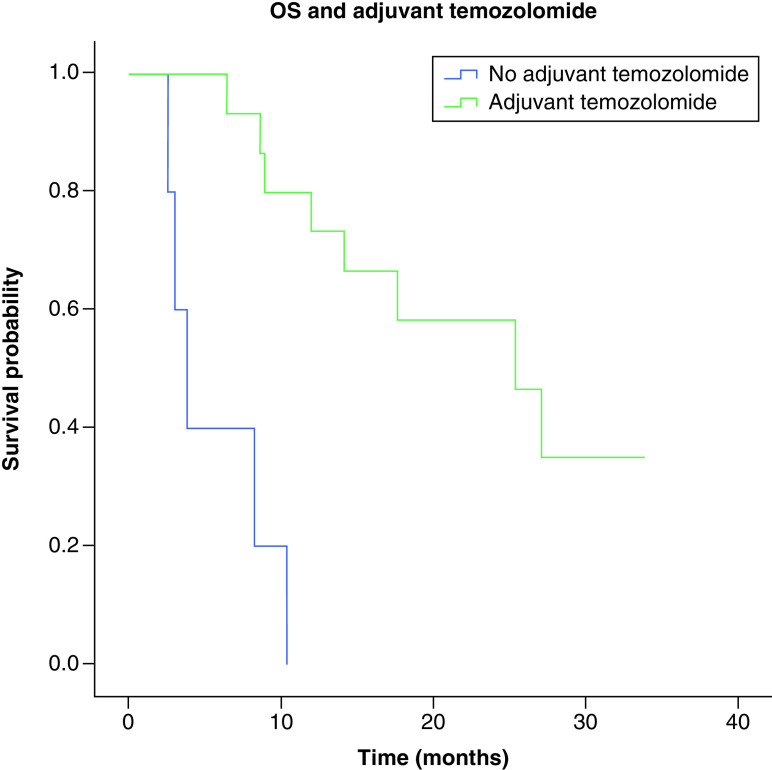
Overall survival and circulating endothelial cells. The median overall survival was 17.4 months for patients in group 1 and 8.12 months for patients in group 2 (p = <0.001). n = 10, group 1; n = 5, group 2. OS: Overall survival.

**Figure 2.**
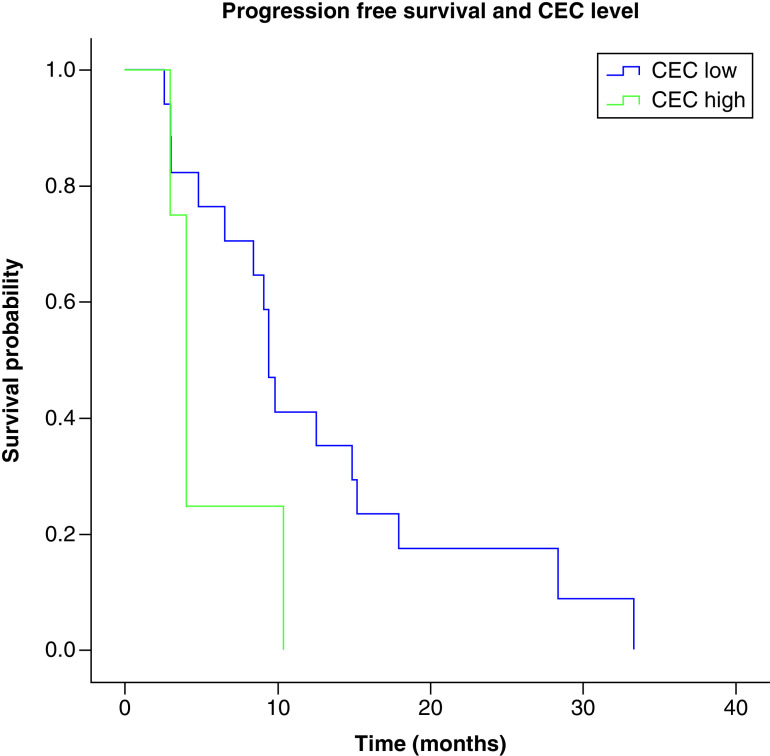
Progression-free survival and circulating endothelial cells. The median progression free survival was 8.94 months for group 1 and 3.95 months for group 2 (p = 0.097). n = 17, group 1; n = 5, group 2. CEC: circulating endothelial cell.

**Figure 3.**
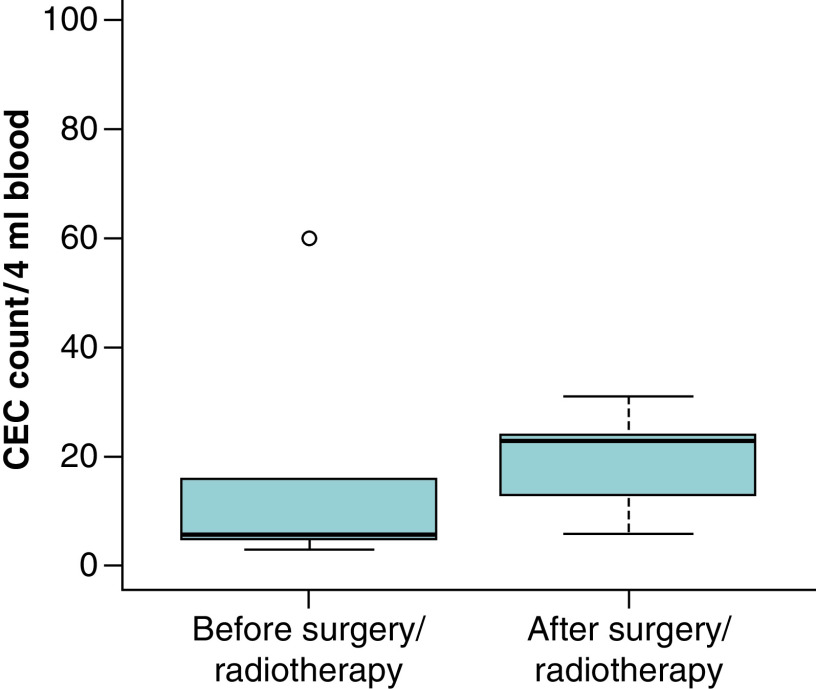
Circulating endothelial cell count before and after surgery and radiotherapy. There was a non-significant increase in CEC count after surgery and radiotherapy, n = 5. CEC: circulating endothelial cell.

The median overall survival of the entire cohort was 11.8 months (range: 3.33–20.32) and was significantly associated with CEC count at diagnosis; 25.33 months (IC 95%: 8.65–42.0093) for patients in group 1 (<40 CEC) and 8.23 months for patients in group 2 (>40 CEC; IC 95%: 4.44–12.0; p = 0.02, Figure 1). Furthermore, the median progression free survival was higher in group 1, 8.94 months (IC 95%: 5.1–12.69) versus 3.95 months (IC 95%: 2.91–4.98) for group 2, but did not reach statistical significance (p = 0.097, Figure 2). This may be due to the limited size of the patient cohort. CEC count at diagnosis and after surgery and radiotherapy was available for five patients, there was an increase in the mean CEC count after surgery and radiotherapy (18 at diagnosis vs 19.4 after surgery and radiotherapy), although the difference did not reach statistical significance (Figure 3).

**Figure 4.**
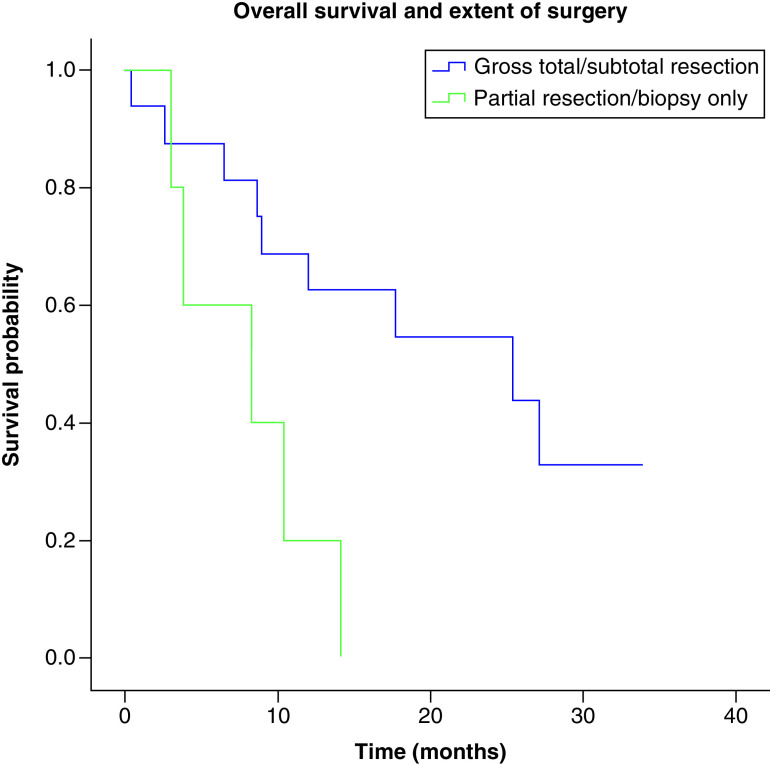
Overall survival and extent of surgery. Median overall survival depending on the extent of surgery was 25.33 months (IC 95%: 5.54–45.12) for complete and subtotal resection and 8.23 months (IC 95%: 0.0–17.75) for partial resection or biopsy only (IC), p = 0.014. n = 16, complete/subtotal resection; n = 6, partial resection/biopsy.

**Figure 5.**
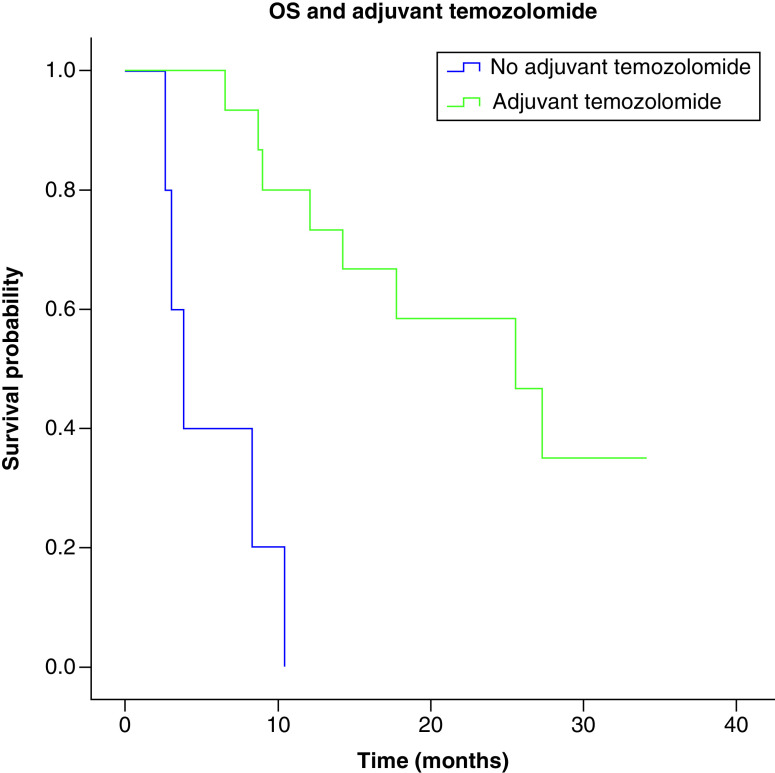
Overall survival and adjuvant temozolomide. Median overall survival depending on treatment with adjuvant temozolomide was 6.936 months for no adjuvant temozolomide or 17.42 months for adjuvant temozolomide (p ≤ 0.001). n = 6, only radiotherapy; n = 15, adjuvant temozolomide. OS: Overall survival.

Median overall survival was significantly associated with the extent of surgery; 25.33 months (IC 95%: 5.54–45.12) for complete and subtotal resection and 8.23 months (IC 95%: 0.0–17.75) for partial resection or biopsy only (IC; p = 0.014) (Figure 4). However, this was not related to CEC count as an equal number of patients (8) from group 1 and group 2 were in the complete/subtotal and partial resection/biopsy group. Median overall was significantly associated with treatment with adjuvant temozolomide; 6.936 months (IC 95%: 0.744–13.12) for no adjuvant temozolomide or 17.42 months (IC 95%: 6.79–28.05) for adjuvant temozolomide, p ≤ 0.001 (Figure 5).

Four patients who were initially considered for the study but whose pathological report did not confirm a glioblastoma were analyzed for CEC. The results were as follows: an oligoastrocytoma with 31 CEC, a low-grade astrocytoma with 4 CEC, an oligodendroglioma with 1 CEC and a recurrent glioblastoma with 4 CEC. One patient had CEC determinations at different stages of the tumor treatment. The patient started with 3 CEC prior to surgery. At the time of finishing radiotherapy there were 31 CEC and they then started temozolomide for 6 cycles and progressed. At this time, there were 1329 CEC and they started bevacizumab. After 9 months, they progressed and at this point the CEC count was 177.

### CEC count & MRI parameters

A Spearman test was performed to determine a possible correlation between CEC and rCBV in the MRI. Spearman's rho was 0.4563 (p = 0.1584). The preoperative CEC were not found to correlate significantly with the tumor rCBV.

## Discussion

CEC represent an attractive conceptual biomarker. Our results demonstrate that CEC can be detected in recently diagnosed glioblastoma patients. We found that the preoperative CEC counts in GB patients were significantly associated with a poorer OS and PFS. This may be related to the increased angiogenic process and its prognostic implications. These results suggest that there may be increased shedding of endothelial cells into the circulation from abnormal blood vessels in cases with the worst prognosis. Some studies have found an association between circulating markers and clinical outcome and showed that CEC pretreatment was associated with OS [15]. However, other studies in other tumor types have shown conflicting results with regard to the association between baseline CEC count and clinical outcome [16,17]. Preoperative CEC number in GB patients have been found to be significantly higher than the CEC number in human controls and it has also been determined that CEC levels decrease significantly in the postoperative period, although these studies did not find any correlation with PFS or OS [18]. In our study, there was an increase in CEC count after surgery and radiotherapy, which may be related to the vascular damage that occurs during targeted treated and has been previously reported [19]. However, other studies have shown a decrease in CEC levels after treatment with RT and concomitant temozolomid in Glioblastoma patients (ref). These differences may be due to the blood sampling time after treatment due to short- and long-term effects on CEC count [15].

An association between positive outcomes and number of CEC has been found in patients with cancer receiving antiangiogenic drugs, but nevertheless, this association has not been found by others [20,21,22,23,24,25]. For example, a decrease in CEC number and low baseline count have previously been correlated with a positive response to bevacizumab [21,22]. However, there was no correlation between CEC level and PFS or OS in a trial of bevacizumab with sorafenib [23]. Other factors may also influence CEC numbers, such as dexamethasone (induction of endothelial inflammation) [24], contrast agents used in MRI scans (influences cell homing to the liver) [25] and epilepsy [26], which may lead to an increase in VEGF levels and affect the CEC number [27]. However, due to the limited size of the patient cohort, these factors and the effect of anti-angiogenic therapy on CEC count could not be accurately assessed in this study.

In this study, we verified the number of CEC after exposure to bevacizumab in one patient. The CEC count was 1329 when he started bevacizumab. After 9 months the patient progressed and at this point the CEC count was 177. In this case, bevacizumab seems to have some effect on peripheral CEC. In this study, the preoperative CEC were not found to correlate significantly with the PWI. Although the highest tumor vascularity detected by rCVB parameter could be expected to correlate with CEC, and other authors have found a correlation [18]. One possible explanation is the low number of patients analyzed that may limit the statistical analysis.

There are several limitations of the study that must be highlighted. First, the sample size is an important limiting factor due to the low frequency of this tumor type. Furthermore, CEC were detected in patients with other tumor types that were initially included in the study due to a suspicion of glioblastoma. This included, an oligoastrocytoma with 31 CEC, a low-grade astrocytoma with 4 CEC, an oligodendroglioma with 1 CEC and a recurrent glioblastoma with 4 CEC. This confirms that CEC are be present in other pathologic entities and are not specific to GB. Another limitation is that there have been a variety of inmunophenotypic definitions for CEC and CEP that may contribute to the conflicting results in the literature. Searching for CTCs in the blood of patients with GB is difficult, given that reliable tumor-specific cell surface markers have yet to be established. As the CellSearch Assay^®^ was used in this study, the CEC phenotype was strictly classified as CD146^+^, CD105^+^ CD45- and DAPI+, in accordance with the US FDA approval. However, other definitions of CEC have included CD45-/CD31bright/CD34^+^; CD45-/CD34^+^/CD146^+^ [4] and CD45-/AE5-/Cd146+ [28]. Other studies in glioblastoma have used CEC definition as CD45-/CD31^+^ cells while CEP were defined as CD31^+^/CD133^+^ [18]. Others have defined CEC as CD34^+^/CD146^+^ CD45- and DRAQ5+ [29] or as CD45^+^/CD34^+^/CD133^+^ [30]. In spite of the limitations, this study provides preliminary data of the usefulness of CEC detection to predict prognosis in GB and highlights their potential use as a treatment response marker.

## Conclusion

CEC count at diagnosis is a potential prognostic marker in glioblastoma patients.

**Summary points**

- Circulating endothelial cells (CEC) determination at diagnosis have a prognostic vale in glioblastoma patients.
- An average CEC count higher than 40 CEC/4 ml blood at diagnosis correlates with a poorer overall survival.
- An average CEC count higher than 40 CEC/4 ml blood at diagnosis correlates with a shorter progression free survival.
- There is no correlation between CEC count and PWI (perfusion-weighted imaging) RM.
- Patients that undergo a complete and subtotal resection have a better overall survival compared with those that undergo a partial resection.
- Adjuvant with temozolomide improves overall survival.
